# Zip Nucleic Acid-Based Genomagnetic Assay for Electrochemical Detection of microRNA-34a

**DOI:** 10.3390/bios13010144

**Published:** 2023-01-15

**Authors:** Arzum Erdem, Ece Eksin

**Affiliations:** 1 Department of Analytical Chemistry, Faculty of Pharmacy, Ege University, Izmir 35100, Turkey; 2 Biomedical Device Technology Program, Vocational School of Health Services, Izmir Democracy University, Izmir 35290, Turkey

**Keywords:** zip nucleic acid (ZNA), miRNA, magnetic beads (MBs), pencil graphite electrodes (PGE), differential pulse voltammetry (DPV)

## Abstract

Zip nucleic acid (ZNA)-based genomagnetic assay was developed herein for the electrochemical detection of microRNA-34a (miR-34a), which is related to neurological disorders and cancer. The hybridization between the ZNA probe and miR-34a target was performed in the solution phase; then, the resultant hybrids were immobilized onto the surface of magnetic beads (MBs). After magnetic separation, the hybrids were separated from the surface of MBs and then immobilized on the surface of pencil graphite electrodes (PGEs). In the case of a full-match hybridization, the guanine oxidation signal was measured via the differential pulse voltammetry (DPV) technique. All the experimental parameters that influenced the hybridization efficiency (i.e., hybridization strategy, probe concentration, hybridization temperature, etc.) were optimized. The cross-selectivity of the genomagnetic assay was tested against two different miRNAs, miR-155 and miR-181b, individually as well as in mixture samples. To show the applicability of the ZNA-based genomagnetic assay for miR-34a detection in real samples, a batch of experiments was carried out in this study by using the total RNA samples isolated from the human hepatocellular carcinoma cell line (HUH-7).

## 1. Introduction

Zip nucleic acid (ZNA) has been described as an oligonucleotide conjugated with repeated spermine units. There is a decrease observed at electrostatic repulsions with target nucleic acid strands since the spermine units of ZNA are cationic, which increases the affinity and specificity of ZNA. The cationic spermine units can be attached to an oligonucleotide from any position. The melting temperature (T_m_) of the duplex structure can be linear, smooth, or predictable [1,2,3,4,5,6].

ZNA oligonucleotides have been used as probes for polymerase chain reaction (PCR) analysis [1,2,4,5,6]. Gagnon and coworkers [5] synthesized ZNA oligonucleotides as anti-sense and anti-gene agents. An anti-sense application was performed to inhibit human huntingtin (HTT), and an anti-gene application was used to block the transcription of the human progesterone receptor. Gagnon and coworkers showed that ZNA oligonucleotides could be used as anti-sense and anti-gene agents. They reported that the DNA anti-sense sequence became a selective and efficient inhibitor for HTT after oligospermine conjugation. In the study of Noir et al. [2], ZNA oligonucleotides were synthesized. It was reported that the T_m_ of ZNA oligonucleotide was predictably dependent on the number of spermine.

Begheldo et al. [6] studied whole mount in situ hybridization (ISH) protocols to target endogenous miRNAs in *Arabidopsis* seedlings using ZNA probes. They showed that the ZNA probe based ISH protocol was a real alternative to the sectioning of different plant species. Furthermore, they reported that ZNA probes were more effective and selective than locked nucleic acids (LNA) for the ISH protocol. For its application to biosensors, ZNA probes were used for the first time in our previous works [7,8,9,10,11]. A ZNA probe was applied in combination with a magnetic bead (MB) assay initially by Erdem and Eksin in 2019 [10] for the electrochemical monitoring of single-nucleotide polymorphisms (SNPs) related to Factor V Leiden mutation. Streptavidin-coated magnetic beads (MBs) were used to prepare the ZNA–DNA hybrid samples. Hybridization was performed on the surface of MBs, and accordingly, the guanine signal was measured as a response to hybridization using carbon nanofibers (CNFs), modified screen-printed electrodes (SPEs), and multichannel screen-printed electrode array (CNF-MULTI SPEx8) as sensing platforms. The application of the ZNA to the voltammetric detection of a single-nucleotide mutation was presented in our previous study by using carbon nanofiber (CNF), modified screen-printed electrodes (SPEs), and a multichannel screen-printed electrode array (MULTIx8 CNF-SPEs) [9]. An impedimetric protocol was performed by means of an efficient ZNA probe for SNP related to FV Leiden. The effective discrimination of single-point mutations, such as G to A, G to C, and G to T, was successfully achieved by using an eight-channel array of electrodes [11].

microRNAs (miRNAs, miRs) are 18–25 nucleotide-long RNAs and have been described as a new class of endogenous RNAs. They are related to several vital human diseases, such as heart failure, cancer, diabetes, vascular disease, and neurological disorders [12,13]. Their roles comprise crucial biological pathways, including proliferation, differentiation, and apoptosis. It was proven in previous works that miRNAs could be used as biomarkers for the diagnosis of cardiovascular diseases [14,15], different types of cancer [16], and neurological disorders [17]. Highly sensitive methods are required to identify extremely low levels of miRNAs in the bloodstream. Since miRNAs may differ by only one base, the method must also be selective. Additionally, this method must meet requirements such as minute sample volume and cost-effectiveness, as well as the multiplexing capabilities of a diagnostic test. Finally, the detection method for point-of-care applications should enable the direct assessment of miRNAs without prior amplification or labeling. In this regard, electrochemical biosensors have recently been used to achieve accurate miRNA detection. The approaches to direct miRNA detection are based on the electrochemical signal and have been reviewed by Aamri et al. in detail [18]. Chen et al. discussed using nanomaterials and oligonucleotides as amplification strategies for miRNA detection [19]. For instance, a label-free electrochemical miRNA detection method, which relied on the target-miRNA-induced reduction of Cu^2+^ and the consequential changes in the electrochemical signals generated from the remaining Cu^2+^, was reported by Kim et al. [20]. An electrochemical biosensor was developed for the simultaneous detection of miRNA-141 and miRNA-21 by Yuan et al. [21]. Two RedOx molecules, Thi and Fc, were attached to the magnetic nanoparticle and the captured probe, leading to the attachment of many RedOx molecules. In the presence of miRNAs, the hybridization chain reaction was performed, and then the DNA1/Fe_3_O_4_NPs/Thi and DNA2/Fe_3_O_4_NPs/Fc were captured by the formed dsDNA. This process generated many magnetic nanoprobes with attachments to the surface. Mandli et al. [22] developed an electrochemical miRNA biosensor based on a sandwich system using AuNPs as a biosensor platform incorporating Streptavidin-linked alkaline phosphatase (SALP) and enzymes linked to a biotin-modified signaling probe, catalyzing α-naphthyl phosphate as a substrate to produce electroactive α-naphthol. The DPV technique was used to measure the α-naphthol oxidation signal. The ionic-liquid-modified chemically activated pencil graphite electrodes were developed for label-free voltammetric detection of miRNA-34a by Yarali et al. [23]. They further implemented this method to the total RNA samples isolated from the HUH-7 human hepatocellular carcinoma cell line for the detection of miRNA-34a.

miR-34a is an important tumor suppressor that inhibits tumor progression and oncogenesis. Numerous studies conducted in recent years have revealed that miR-34a has low expression in a range of carcinomas with the loss of its tumor-suppressing ability [24]. Thus, miR-34a is crucial for invasion, metastasis, and proliferation. Many researchers have examined the prognostic value of miR-34a in a variety of malignancies, such as head and neck squamous cell carcinoma, thyroid neoplasms [24], gastric [25], colorectal [26], and breast cancer [27]. Therefore, miR-34a is considered to be a potential tumor marker. On the other hand, growing evidence indicates that the overexpression of the miR-34 family in the brain may play a crucial role in Alzheimer’s disease pathogenesis by targeting and downregulating the genes associated with neuronal survival, synapse formation and plasticity, mitochondrial function, and energy metabolism [28].

Hepatocellular carcinoma (HCC) is the most common type of primary liver cancer. Recent studies have shown that the expression of miR-34a is dramatically decreased in clinical HCC specimens, suggesting that miR-34a represents a potential target for HCC treatment [29]. The expression level of miR-34a in six HCC cell lines (Huh7, HCCLM3, HepG2, Hep3B, Mahlavu, and SNU475) and the human hepatocyte line L02 was studied by Zhang et al. [29], and it was found that the expression of miR-34a was downregulated in HCC cell lines and clinical specimens. However, the biological effect and underlying mechanism of miR-34a in HCC tumorigenesis and metastasis remain to be elucidated.

To the best of our knowledge, no report presenting a ZNA-based electrochemical biosensing assay developed for the detection of any microRNAs in combination with magnetic beads (MBs) exists in the literature. In the present study, the aim was to develop a novel genomagnetic assay for the electrochemical monitoring of miR-34a, which is related to Alzheimer’s disease [17] and cancer [16]. For this purpose, a ZNA probe, which was complementary to miR-34a, was used to increase the effectiveness of nucleic acid hybridization. Firstly, hybridization occurred between the biotinylated ZNA probe and miR-34a, and the hybrids immobilized on the streptavidin-coated magnetic beads (MBs) surface through biotin/streptavidin interaction. After magnetic separation steps, the resultant hybrids were separated from MBs and then immobilized onto the surface of pencil graphite electrodes (PGEs). Accordingly, the measurement of the guanine oxidation signal was performed via the differential pulse voltammetry (DPV) technique. The selectivity of the ZNA-based-genomagnetic assay was then tested with non-complementary (NC) miRNAs; miR-155 and miR-181b. This study provides the first results presenting the potential use of ZNA probes for the selective and sensitive electrochemical detection of microRNA in the literature. 

## 2. Materials and Methods

### 2.1. Instruments

The measurement of the guanine oxidation signal by using disposable pencil graphite electrodes (PGEs, Tombow 0.5, HB,) was performed by using µAUTOLAB PGSTAT via the differential pulse voltammetry (DPV) technique. The magnetic bead (MB) assay was performed by using a magnetic separator, MCB1200 (Sigris, Brea, CA, USA). 

The Supplementary Materials section provides more information related to the instruments and chemicals used in this study.

### 2.2. Chemicals

The biotinylated DNA probe and miR-34a RNA oligonucleotide (ODN) were acquired from Ella Biotech (Germany), and the biotinylated ZNA probe was acquired from MetaBion (Steinkirchen, Germany). 

The DNA probe 5′-biotin-ACAACCAICTAAIACACTICCA-3′ (I: inosine) and 5′ end biotin linked ZNA probe 5′-bio-5S-ACAACCAICTAAIACACTICCA-3′ (S: Spermine) used as a capture probe for miRNA-34a target sequence (5′-UGGCAGUGUCUUAGCUGGUUGU-3′) (U: Uracil). The selectivity of the assay was tested in the presence of other miRNAs as miR-155 (5′-UUAAUGCUAAUCGUGAUAGGGGU-3′) and miR-181b (5′-AACAUUCAUUGCUGUCGGUGGGU-3′).

Briefly, a 500 µg/mL stock solution of the DNA probe was prepared in 10 mM Tris–EDTA (TE) buffer containing 1 mM EDTA (pH 8.00). A 475 µg/mL stock solution of the ZNA probe was prepared in 2.5xDubbelco’s PBS (5.40 mM KCl, 3 mM KH_2_PO_4_, 273.80 mM NaCl, 17.80 mM K_2_HPO_4_, pH 7.40). A 500 µg/mL stock solution of the RNA target was prepared in ultrapure water. All ODNs were aliquoted and kept frozen at −20 °C. ODNs were diluted in 50 mM phosphate-buffered saline (PBS, pH 7.40).

Streptavidin-coated magnetic particles (magnetic beads; MBs) 0.94 mm in diameter were purchased from Estapor, Merck (France). Other chemicals were in analytical reagent grade, and they were supplied by Sigma and Merck.

### 2.3. Genomagnetic Assay

The solution-phase hybridization assay between the 4 µg/mL ZNA probe and miR-34a target sequence was carried out by mixing these sequences in 20 µL PBS (pH 7.40) under gentle mixing for 10 min.

In the meantime, 3 µL of MBs were transferred into a centrifuge tube and washed with TTL buffer (100 mM Tris–HCl, 0.1% Tween 20 and 1 M LiCl, pH: 8.00), followed by a secondary washing step with PBS (pH 7.40) for 5 min [30,31,32,33,34,35,36]. Then, a 20 µL sample containing the ZNA–miRNA hybrid was incubated with MBs for 30 min with gentle mixing. Hybrid immobilized MBs were separated and washed twice with PBS (pH 7.40) and resuspended in 0.05 M NaOH for alkaline treatment for 5 min, followed by dilution in ABS (pH 4.80) as the last step of sample preparation. PGEs were dipped into the sample and kept for 15 min, and the ZNA–miRNA hybrid physically adsorbed onto the PGE surface without using any chemical linker agent or covalent attachment step. Then, each PGE was washed with 0.5 M ABS (pH 4.80), to eliminate non-specific binding [30,31,32,33,34,35,36], followed by electrochemical measurement. The guanine oxidation signal was measured as a result of a successful hybridization event. 

**Scheme 1:**
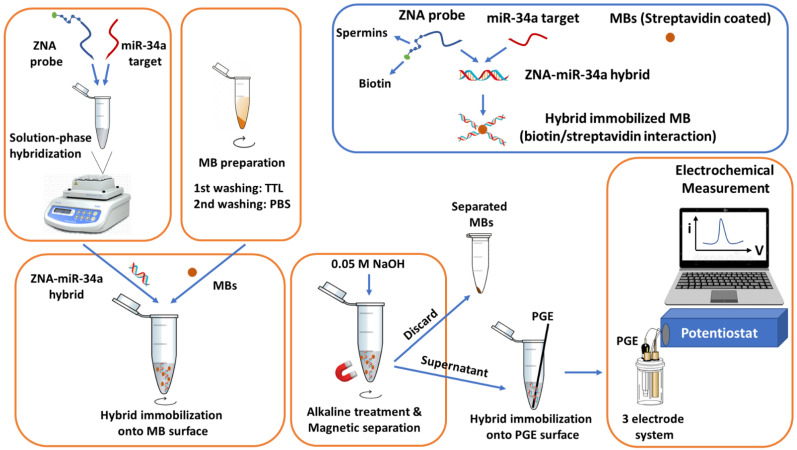
The schematic view of ZNA probe based genomagnetic assay used for electrochemical sensing of miRNA-34a by using PGE.

Since the ZNA probe contains inosine instead of guanine bases, direct electrochemical detection was possible by measuring the guanine oxidation signal in case of a successful hybridization event [37,38,39,40,41]. The detection protocol is depicted in Scheme 1.

### 2.4. Isolation of Total RNA from Cell Lysates

Total RNA Purification Kit (Norgen, Thorold, ON, Canada) was used to isolate the total RNA from the HUH-7 human hepatocellular carcinoma cell line according to the manufacturer’s instructions. RNA concentrations were determined by measuring the absorbance at 260 nm with a spectrophotometer (Nanovette, Beckman Coulter, Brea, CA, USA). The isolation protocol of the total RNA from the HUH-7 human hepatocellular carcinoma cell line was performed according to the protocol given in our previous work [23].

## 3. Results and Discussion

**Figure 1:**
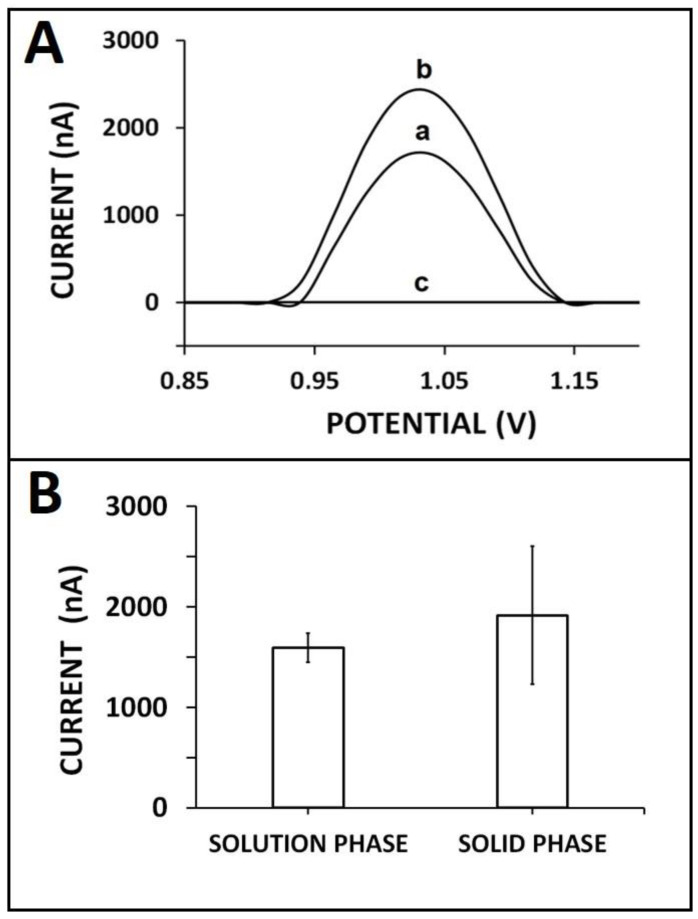
The comparison of different hybridization strategies: (**A**) voltammograms and (**B**) histograms representing guanine oxidation signal after hybridization of 4 µg/mL ZNA probe and 20 µg/mL miRNA-34a target using different hybridization strategies: (a) solution-phase hybridization, (b) solid-phase hybridization, and (c) the control experiment in the absence of miRNA-34a.

Firstly, the efficiency of the ZNA–miRNA hybridization was tested by comparing different hybridization strategies such as solid-state hybridization and solution-phase hybridization. These strategies were widely used by our group in previous works [23,42]. Since this was the first ZNA-probe-based genomagnetic assay for miRNA analysis, the effect of the hybridization strategy was an essential step in the first experiment of this work. For this purpose, step-by-step hybridization was performed by immobilizing the biotin-linked ZNA probe onto the MB–STR surface. After the probe immobilization step, the miR-34a target sequence was added, and hybridization was performed onto the surface of MB–STRs for 60 min by gentle mixing. On the other hand, solution-phase hybridization was performed by mixing the ZNA probe and the complementary miRNA target. The samples were allowed to hybridize by gentle mixing for 10 min. Then, ZNA–miRNA hybrids were incubated with MBs by gentle mixing for 30 min, as mentioned in the experimental section. The rest of the experimental steps were the same for each hybridization procedure. The guanine oxidation signals were measured as 1916.50 ± 688 nA (relative standard deviation (RSD)% = 35.90%, *n* = 2) and 1592 ± 142 nA (RSD% = 8.9%, *n* = 2) with solid-phase and solution-phase hybridization procedure, respectively (Figure 1). 

In the case of solid-phase hybridization, the highest guanine oxidation signal was obtained; nevertheless, the relative standard deviation (RSD%) was too high (RSD%, 35.90%), which makes these results statistically insecure. This may indicate that the hybridization efficiency was low due to the diffusion barrier that ensued during solid-phase hybridization, which was mentioned by Söderlund et al. [43]. Inefficient hybridization usually occurs due to steric hindrance and surface electrostatic forces, which affect the ability of the target to properly find its capture probe [44]. Consequently, solution-phase hybridization was preferentially used to achieve more efficient hybridization in the present work.

**Figure 2:**
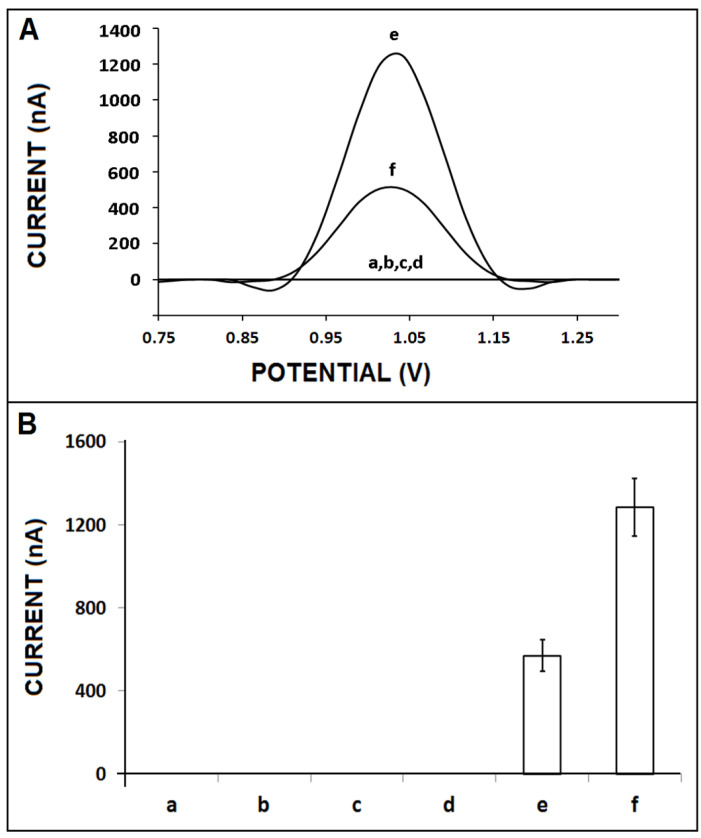
The effect of the ZNA probe on the performance of nucleic acid hybridization: (**A**) DPVs and (**B**) bar graphs representing the control signal of (a) spermine, (b) DNA probe, (c) ZNA probe, and (d) after the interaction of spermine and 10 µg/mL miR-34a target. The average guanine signals (*n* = 3) were obtained after hybridization of 10 µg/mL miR-34a target with (e) DNA probe and (f) ZNA probe.

Next, the effect of the ZNA probe on the performance of nucleic acid hybridization was tested. A control experiment was also performed to investigate the interference effect of spermine. For this purpose, hybridization was performed between DNA probe/ZNA probe/spermine individually with miR-34a target. In the case of interaction between spermine and miR-34a target (Figure 2(A,B-d)), there was no signal measured. The average guanine signals were measured as 569.8 ± 77.2 nA (RSD%, 13.6%, *n = 3*) and 1283.3 ± 139.4 nA (RSD% = 10.9%, *n* = 3), respectively, in the case of the hybridization of DNA probe/ZNA probe with 10 µg/mL miR-34a target (Figure 2(A,B-e,f)). This signal enhancement was strong proof of the effectiveness of the ZNA probe in the hybridization process, and its zipped structure at the 5′ end was opened during hybridization. Additionally, the backbone of ZNA was more positive due to its spermine groups, in contrast to the DNA probe. Therefore, the cationic nature of the ZNA probe made it easy to capture and hybridize to its target sequence effectively. The ZNA probe improved the hybridization efficiency as a result of overcoming the electrostatic repulsion between the probe and its complementary sequence [2]. Thus, a higher guanine signal was recorded herein after the hybridization of the ZNA probe with its miRNA-34a target. 

**Figure 3:**
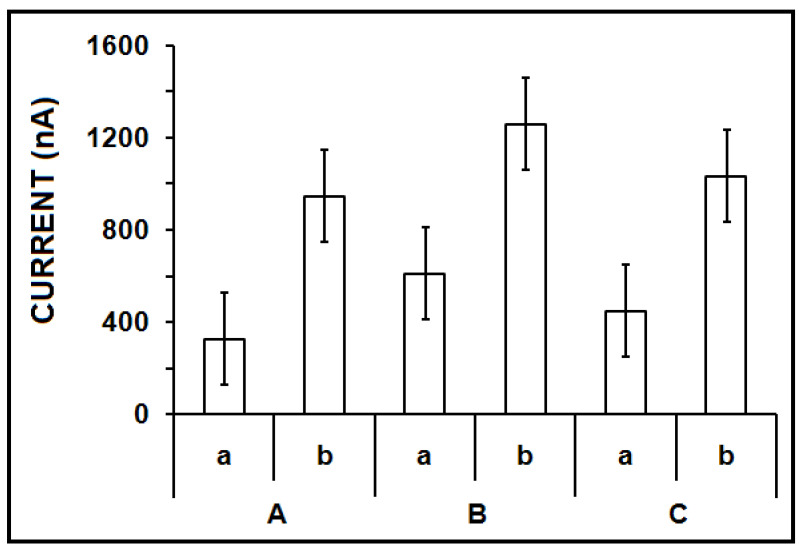
The effect of the temperature on the hybridization between DNA or ZNA probe and miR-34a target. Bar graphs representing the average guanine signals (*n* = 3) obtained after hybridization of (a) DNA probe with miR-34a target, (b) ZNA probe with miR-34a target at (**A**) 25 °C, (**B**) 50 °C, and (**C**) 75 °C hybridization temperature.

Next, the effect of the temperature in the hybridization process was tested. The hybridization between the ZNA or DNA probe and miR-34a target was performed at different temperatures, and the guanine signals for each case were evaluated (Figure 3). In the presence of the ZNA probe, there was a 1.91-, 1.27-, and 1.31-fold increase at the guanine signal in comparison to the ones obtained with the DNA probe at 25 °C, 50 °C, and 75 °C, respectively. The highest and most reproducible guanine signal was recorded at 50 °C (Figure 3(B-b)) as 1297 ± 141.9 nA, with the RSD% as 10.9% (*n* = 3). This result may be attributed to the melting temperature (T_m_) of the ZNA probe and its miR target (i.e., 53 °C). A more stable Watson–Crick duplex could be formed at 50 °C [2] by using the ZNA probe (Figure 3(B-b)) in comparison to the one obtained by the DNA probe (Figure 2(B-a)). Thus, 50 °C was chosen as the optimum hybridization temperature for further experiments in our study.

**Figure 4:**
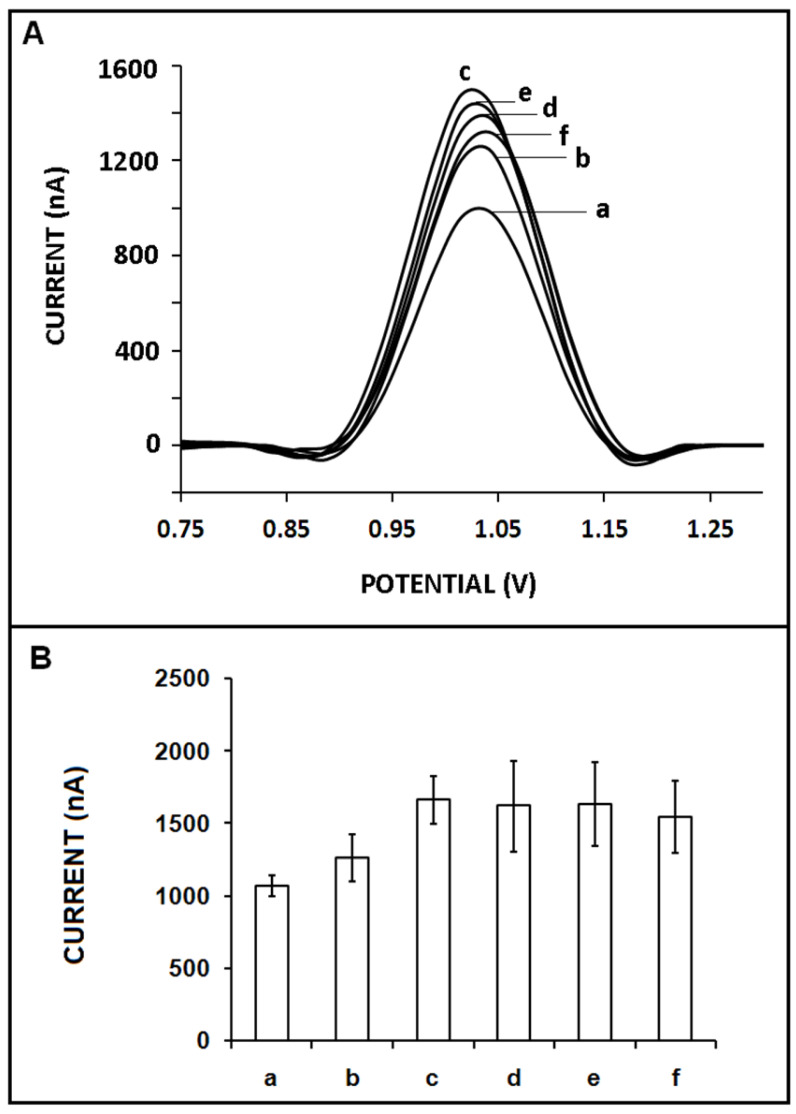
The effect of ZNA probe concentration on the hybridization efficiency: (**A**) voltammograms and (**B**) bar graphs representing the average guanine signals recorded after the hybridization of ZNA probe at the concentration level of 1 to 10 µg/mL and 10 µg/mL miR-34a target. The average guanine signals (*n* = 3) recorded after hybridization between (a) 1, (b) 2, (c) 4, (d) 6, (e) 8, and (f) 10 µg/mL ZNA probe and 10 µg/mL miR-34a target.

The effect of the concentration of the ZNA probe on the sensor response was explored (Figure 4). The hybridization of the 10 µg/mL miR-34a target and the ZNA probe with its different concentrations varying from 1 to 10 µg/mL was performed. There was an increase in the guanine signal when the ZNA probe’s concentration increased to 4 µg/mL, and it was measured as 1660 ± 164.6 nA (RSD%= 9.9%, *n* = 3), which was the highest guanine signal (Figure 4(A,B-c)). At higher probe concentrations, the guanine signal quantity did not significantly alter; nevertheless, the RSD % was too high in the presence of higher ZNA probe concentrations, which makes these results statistically insecure (Figure 4(B-d–f)). Our results demonstrated that a better hybridization efficiency was observed with a 4 µg/mL ZNA probe since the highest and most reproducible guanine signal was recorded. Thus, it was chosen as the optimum ZNA probe concentration in our further experiments.

**Figure 5:**
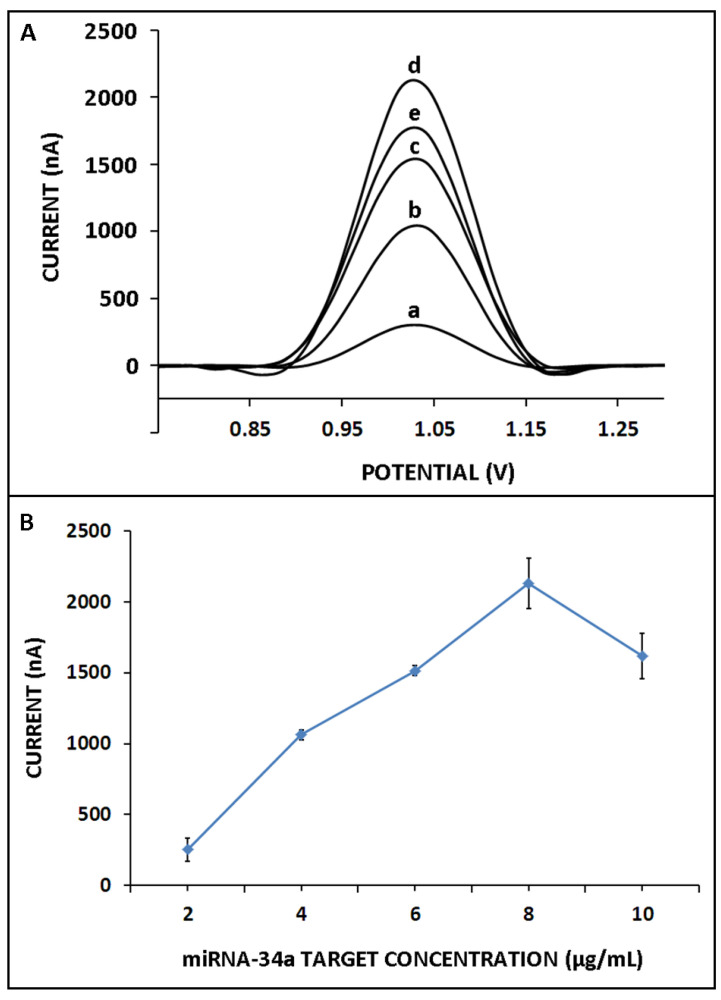
The effect of miR-34a target concentration on the hybridization efficiency: (**A**) voltammograms representing the guanine oxidation signal in case of hybridization between 4 µg/mL ZNA probe and (a) 2, (b) 4, (c) 6, (d) 8, and (e) 10 µg/mL miR-34a target; (**B**) line graph based on average guanine signals that measured after hybridization of 4 µg/mL miR-34a ZNA probe with miR-34a RNA target.

**Figure 6:**
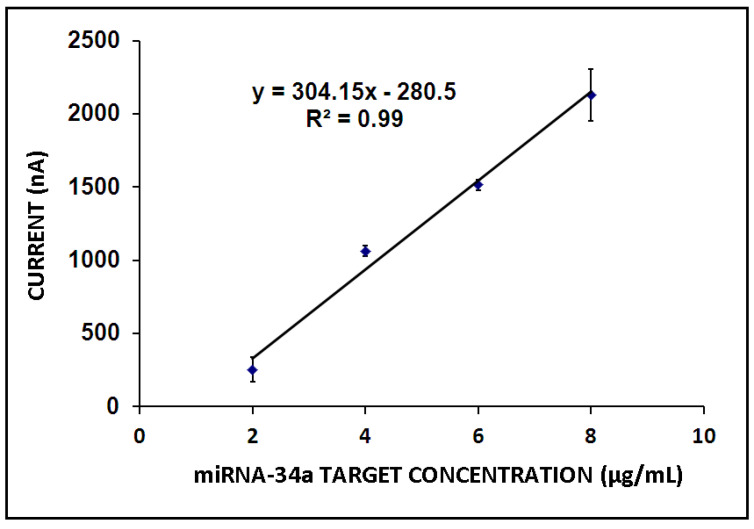
Calibration plot representing the average guanine signal (*n* = 3) in the presence of the hybridization between 4 µg/mL ZNA probe and its miRNA-34a target at different concentration levels ranging from 2 to 8 µg/mL.

Next, the effect of miR-34a target concentration on the hybridization efficiency was examined. Hybridization was performed between the 4 µg/mL ZNA probe and miR-34a in its different concentrations ranging from 2 to 10 µg/mL (Figure 5). There was a gradual increase in the guanine oxidation signal until 8 µg/mL miR-34a, and then, it decreased in the presence of higher target concentrations (Figure 5A,B). Hybridization efficiency was determined under the conditions in which the maximum binding was expected and that provide information about probe accessibility. A hybridization efficiency of 100% indicates that all probe molecules are available for binding to their target molecules, while a lower efficiency suggests that some of the probe molecules may be unable to perform this function [45]. Hence, in the presence of a higher target concentration in comparison to the optimum one, a decrease in the guanine signal was expected herein due to a decrease in hybridization efficiency since the target molecules in higher concentrations may hinder hybridization or negatively affect the conditions for hybridization efficiency. The detection limit (DL) was estimated as 0.87 µg/mL (12.73 pmol in 110 µL sample) by using the equation y = 304.15x − 280.30 (*R*^2^ = 0.99) (Figure 6) [46]. 

**Figure 7:**
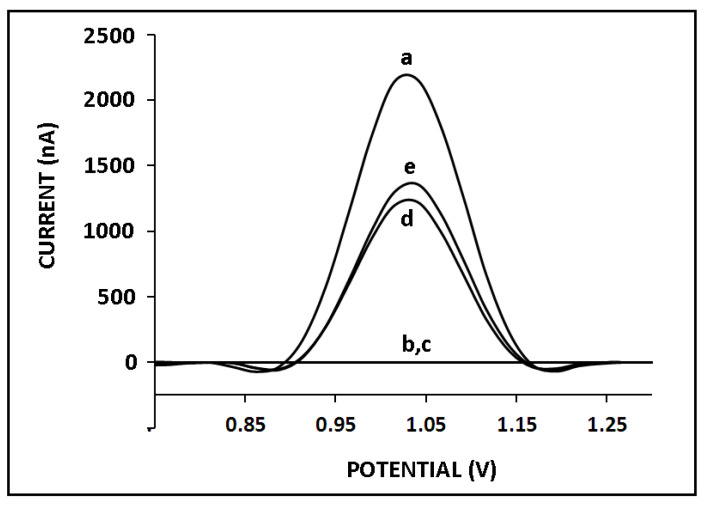
The selectivity of our genomagnetic assay. The guanine oxidation signals in case of hybridization of 4 µg/mL ZNA probe with 8 µg/mL (a) miR-34a RNA target, (b) miR-155, (c) miR-181b, (d) miR-34a and miR-155 mixture, and (e) miR-34a and miR-181 mixture.

The development of nucleic acid biosensors for miRNA analysis requires reliable, highly sensitive, and selective systems that can detect even single-base differentiation since miRNAs might differ from each other by only one base. Therefore, the selectivity of our genomagnetic assay was tested against two different miRNAs (miR-155 and miR-181b). Since the highest guanine signal was measured at 8 µg/mL miR-34a, the selectivity of the sensor was then tested in the presence of 8 µg/mL non-complementary (NC) miRNAs, such as miR-155 and miR-181b (Figure 7). After hybridization occurred between the ZNA probe and miR-34a target, the average guanine signal was measured as 2130 ± 177 nA with the RSD%, 8.34% (*n* = 3), whereas no guanine signal was observed after hybridization with miR-155 (Figure 7b) or miR-181b (Figure 7c). Therefore, it can be concluded that our genomagnetic assay exhibited excellent selectivity against different miR sequences while using the ZNA probe. Subsequently, hybridization between the ZNA probe and miR-34a was performed in the presence of miR-155 or miR-181b (mixture samples), and the guanine signals were slightly lower than the one obtained with full-match hybridization. The guanine oxidation signals were measured as 1225 ± 77.78 nA (RSD%, 6.35%, *n* = 2) and 1330 ± 127 μA (RSD%, 9.57%, *n* = 2) after hybridization between the ZNA probe and miR-34a in the presence of miR-155 or miR-181b. In the mixture samples, the hybridization efficiency may be influenced by other miRNAs. They may hinder hybridization or negatively affect the conditions for hybridization efficiency. Therefore, it was expected that the presence of various miRNA sequences would result in a low guanine signal as well as lower hybridization efficiency. It was concluded that the selectivity of our genomagnetic assay in combination with the ZNA probe to its target miRNA was very good, in contrast to different miRNA sequences. 

To test the applicability of our assay toreal samples, miR-34a detection was performed using the total RNA samples isolated from the HUH-7 cell line. The average guanine oxidation signal was measured as 448 ± 70.17 nA (RSD%, 15.66%, *n* = 3) in the presence of 10 µg/mL total RNA (Figure S1b), whereas there was no oxidation signal in the absence of the total RNA (Figure S1a). The sequence-selective hybridization between the existing miR-34a sequence in the total RNA sample and the ZNA probe successfully occurred, and a guanine signal was obtained (Figure S1). As a result, it was obvious that our ZNA-based genomagnetic assay could detect miR-34a in total RNA samples.

**Figure 8:**
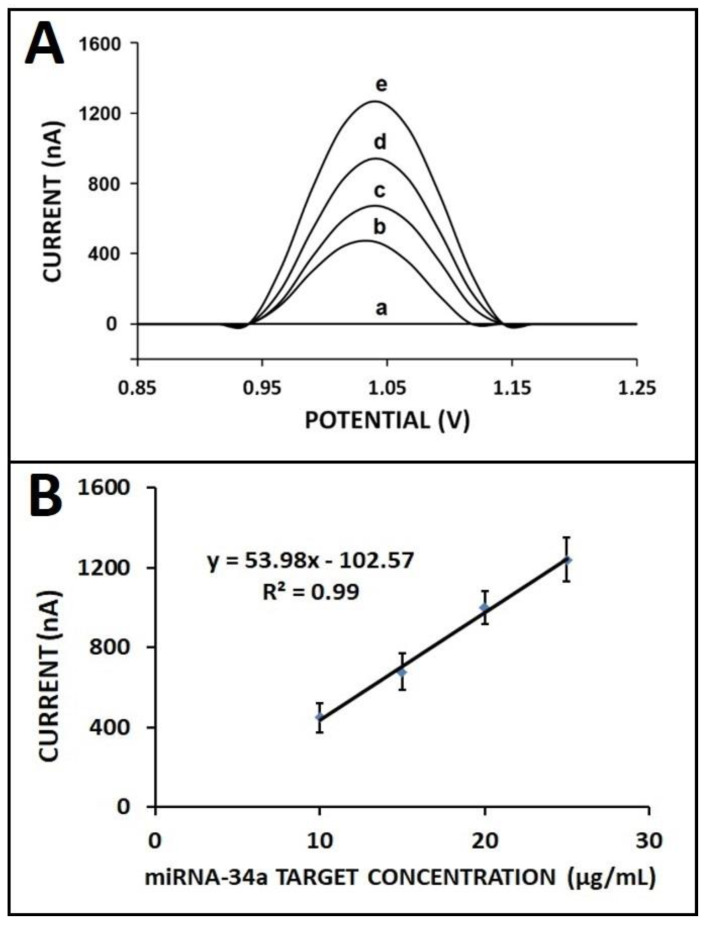
The effect of total RNA concentration on the hybridization efficiency: (**A**) DPVs related to the guanine oxidation signal (a) in the absence of total RNA, after hybridization between ZNA probe with (b) 10, (c) 15, (d) 20, and (e) 25 µg/mL of total RNA sample isolated from HUH-7 cell line; (**B**) calibration plot with the average guanine oxidation signals (*n* = 3).

The effect of the total RNA concentration on sensor response was studied in various concentrations of total RNA samples, ranging from 10 to 30 μg/mL (Figure 8). There was a gradual increase obtained in the case of increased total RNA concentration from 10 to 25 µg/mL, and then the response leveled off in the presence of 30 µg/mL (Figure S2). The detection limit was achieved as 1.25 µg/mL according to the regression equation of y = 53.98x − 102.57 with R^2^ as 0.99.

## 4. Conclusions

The present study aimed to develop a novel genomagnetic assay based on the ZNA probe for the electrochemical monitoring of miR-34a, which is related to neurological disorders and several cancer types. Streptavidin-coated MBs were used for the preparation of the samples containing ZNA–miRNA hybrids. Hybridization was performed in the solution phase, and accordingly, the guanine signal was measured as a response to hybridization using pencil graphite electrodes. ZNA probe was used herein to increase the effectiveness of the nucleic acid hybridization similar to that reported in our previous work [10]. However, the type of target nucleic acid (DNA vs. miRNA), electrode type (SPE vs. PGE), and the hybridization strategy (MB surface vs. solution phase) are the main differences between our earlier study [10] and this present work. The most innovative aspect of our study is that the ZNA probe was applied for the first time in miRNA analysis. No report has been available yet on miRNA detection by using electrochemical or any type of biosensor based on zip nucleic acid (ZNA) as a capture probe.

Under the optimum experimental parameters, the DL was found to be 0.87 µg/mL (12.73 pmol). The cross-selectivity of the genomagnetic assay was tested against different miRNAs, and an excellent selectivity was exhibited against miR-155 and miR-181b, individually as well as in the mixture samples. The hybridization was efficiently performed using the ZNA probe, and the selective genomagnetic assay was successfully performed even in the presence of different miRNA sequences. No guanine signal was observed after hybridization with different miRNAs, such as miR-155 and miR-181b. These results indicated that the developed genosensor was highly specific to miRNA-34a in comparison to the earlier reports [23,42,47,48,49,50,51,52]. Moreover, miR-34a was detected in a relatively short time (i.e., 60 min), which makes our genomagnetic assay a practical method in comparison to other biosensor studies related to miRNA detection in the literature [30,31,53,54]. The implementation of our assay to real sample analysis was also investigated by using the samples obtained from the total RNAs isolated from the HUH-7 cell line. The results of this study showed that our genomagnetic assay was successful enough to carry out miRNA analysis from cancer cell lines while presenting the applicability of our genomagnetic assay to clinical samples. 

ZNA is a new-generation nucleic acid developed for cutting-edge research and innovation in the field of nucleic acid biosensors, specifically SNPs, due to its unique hybridization characteristics concerning its affinity with specificity. Novel biosensing platforms will be developed further by incorporating ZNA into these platforms for their implementation in the PoC system.

## Data Availability

The data presented in this study are available within the article and its Supplementary Materials. Other data that support the findings of this study are available upon request from the corresponding author.

## Supplementary Materials

The following supporting information can be downloaded at: https://www.mdpi.com/article/10.3390/bios13010144/s1. Figure S1. miR-34a detection in total RNA isolated from HUH-7 cell line. DPVs related to the guanine signal (a) in the absence, (b) in the presence of 10 μg/mL total RNA sample isolated from HUH-7 cell line. Figure S2. Line graph presenting the average guanine oxidation signal measured in the case of hybridization of 4 μg/mL ZNA probe with total RNA samples in its various concentrations.
