# Supplementary material for: Zip Nucleic Acid-Based Genomagnetic Assay for Electrochemical Detection of microRNA-34a
Source: Biosensors (Basel). 2023 Jan 15;13(1):144. doi: 10.3390/bios13010144 (PMC9856502; doi:10.3390/bios13010144)
Supplement: Supplementary file 1 [file biosensors-13-00144-s001.zip › biosensors-2032745-supplementary.pdf]

Supporting Information

Zip Nucleic Acid-Based Genomagnetic Assay for Electrochemical Detection of microRNA-34a

Instruments:

μAUTOLAB PGSTAT electrochemical analysis system supplied with GPES 4.9007 software package (EcoChemie, The Netherlands) was used for all of the measurements. The raw data treated using Savitzky and Golay filter (level 2) of software, followed by the moving average baseline correction with a peak width of 0.03.

The three-electrode system consisted of working electrode (PGE), reference electrode (Ag/ AgCl/ 3 M KCl, Model RE-5B; BAS, W. Lafayette, IN) and auxiliary electrode (platinum wire). Tombow pencil was used to hold graphite leads. Electrical contact with the lead was provided by soldering a metallic wire to the metallic part. The lead was held vertically with 14 mm of the lead extruded outside (10 mm of which was immersed in the solution). The pretreatment of the disposable PGEs was performed by applying +1.40 V for 30s by DPV technique. 0.50 M acetate buffer containing 20 mM NaCl (ABS, pH 4.80) was used as electrolyte for pretreatment step.

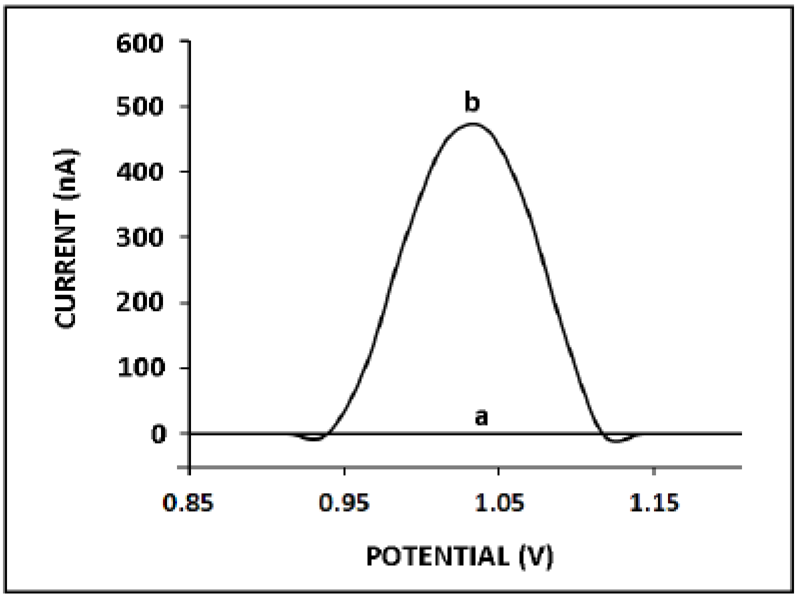

Figure S1. miR-34a detection in total RNA isolated from HUH-7 cell line. DPVs related to the guanine signal (**a**) in the absence, (**b**) in the presence of 10 μg/mL total RNA sample isolated from HUH-7 cell line.

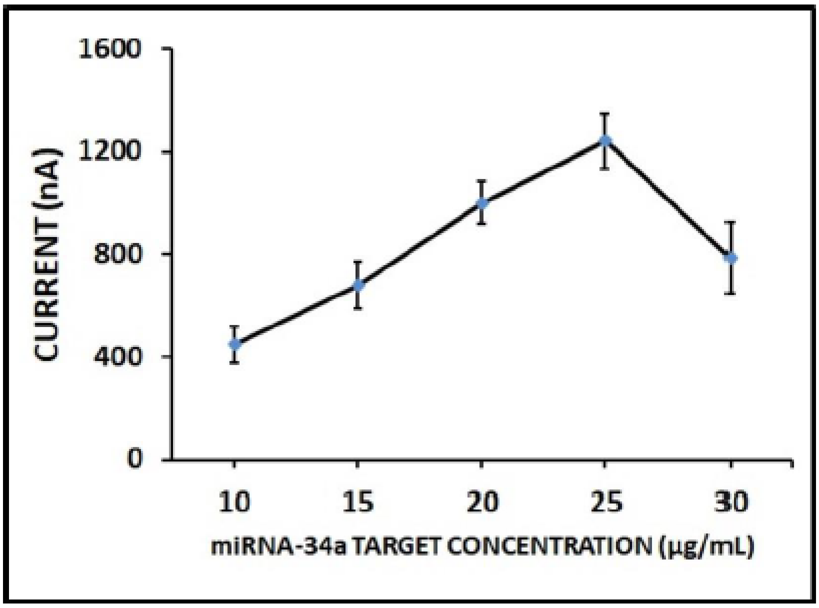

Figure S2. Line graph presenting the average guanine oxidation signal measured in the case of hybridization of 4 µg/mL ZNA probe with total RNA samples in its various concentrations.
